# Medical Science Liaisons in Clinical Trials for Plastic Surgery: A Scoping Review

**DOI:** 10.1055/s-0045-1813038

**Published:** 2025-12-30

**Authors:** Samuel Dyer, Noelle Garbaccio, Jade Smith, Jose A. Foppiani, Angelica Hernandez Alvarez, Abdulrhman Khaity, Khaled Albakri, Otakar Raska, Samuel J. Lin

**Affiliations:** 1 Medical Science Liaison Society, Miami, Florida, United States; 2 Division of Plastic and Reconstructive Surgery, Beth Israel Deaconess Medical Center, Harvard Medical School, Boston, Massachusetts, United States; 3 Faculty of Medicine, Charles University, Prague, Czech Republic; 4 Faculty of Medicine, Elrazi University, Khartoum, Sudan; 5 Faculty of Medicine, The Hashemite University, Zarqa, Jordan

**Keywords:** Medical Science Liaison, plastic surgery, Medical Affairs, clinical research, scientific communication

## Abstract

Medical Science Liaisons (MSLs) serve a pivotal role in bridging scientific research with clinical practice. While their contributions to clinical trials in various medical fields are well documented, their involvement in plastic surgery clinical trials remains underexplored. This scoping review aimed to assess the extent to which MSLs are acknowledged in U.S.-based plastic surgery clinical trials and evaluate their potential impact on trial quality and outcomes. Following PRISMA guidelines, a systematic search was conducted across PubMed, Scopus, Cochrane Library, and ClinicalTrials.gov through June 2025. Clinical trials in plastic and reconstructive surgery were included if they mentioned MSLs in authorship or acknowledgments. Data extraction and quality assessment were conducted independently by two reviewers using a predesigned tool and the NIH quality assessment tool, respectively. Of 3,766 identified studies, only two trials met the inclusion criteria. Both involved MSLs either as coauthors or acknowledged contributors. These studies evaluated breast implant safety and postoperative analgesia. MSLs contributed scientific insight, facilitated cross-stakeholder communication, and supported protocol adherence and data interpretation. Despite limited explicit acknowledgment, this review reveals MSLs' strategic involvement in plastic surgery trials. Their inclusion in the research process enhances data quality, scientific communication, and alignment with regulatory standards. These findings support the broader recognition of MSLs in clinical trial infrastructure within plastic surgery.

## Introduction

Plastic surgery is an established field of surgery that includes various procedures to restore or improve form and function. 1 2 Clinical trials are vital in this area of medicine, as they rigorously evaluate the safety and effectiveness of new surgical techniques, devices, and materials. Successful trials depend on clear communication and collaboration among clinical investigators, sponsoring companies, and health care professionals (HCPs). 1 2

Medical Science Liaisons (MSLs) work within the Medical Affairs department, and their primary responsibility is to facilitate the exchange of complex scientific information. 3 4 This exchange enhances the application of clinical research into clinical practice while serving as unbiased medical resources and ensuring that physicians and other HCPs have access to accurate scientific information. 4 Despite their central role in scientific communication, MSLs are often underrepresented in clinical trial literature, particularly in surgical specialties like plastic surgery. This scoping review seeks to address that gap. 4 5 Despite the growing recognition of MSLs within pharmaceutical companies, their contributions to clinical research, especially within surgical disciplines, remain underreported in the literature.

MSLs translate detailed scientific data into important medical insights, ensuring there is a connection between theoretical research and clinical application. 4 5 In addition, their impact is not limited to the phase IV support, as they support the development of comprehensive medical strategies that can significantly influence how the study is conducted and the final outcomes. 4 5 6 Analyzing MSL activities reveals a broad range of responsibilities related to clinical trial research, including supporting company-sponsored trials from Phase I to IV, as well as investigator-initiated studies. 4 A global survey revealed that 66% of USA-based MSLs are involved in supporting and coordinating company-sponsored clinical research, and 74% support investigator-initiated studies. 4 This extensive involvement and support emphasize the critical role MSLs contribute to clinical trial research.

Given the complexity of clinical trials, collaboration with scientifically trained professionals such as MSLs is essential to ensure the accuracy and relevance of scientific communication. USA-based MSLs are highly educated, with most holding doctorate degrees in science, including PharmDs (44%), PhDs (35%), other doctorate degrees (7%), and MDs (5%). 3 4 The diverse scientific backgrounds of MSLs provide the expertise needed to strengthen the scientific rigor of clinical research.

Although the potential benefits of MSL involvement are promising, there are limited published data on their value and impact in plastic surgery clinical trials. MSLs offer valuable scientific updates, clinical insights, and support. These are particularly important in plastic surgery, where clinical trials are critical for assessing devices, materials, and new surgical techniques. Contributions from MSLs can significantly improve the quality and reliability of clinical trial results, as demonstrated by the fact that 83% of HCPs reported that scientific and medical device updates, along with clinical trial data during discussions with MSLs, were most valuable. 4 This scoping review attempts to fill part of the gap in published literature by focusing on two main questions: (1) To what extent are MSLs recognized in plastic surgery clinical trial publications within the USA? (2) What is the value and impact of MSLs on these trials? This scoping review will summarize the publications and acknowledgments of MSLs in surgical clinical trials, helping to explain the extent of their involvement and their prospective role in advancing plastic surgery practices and improving patient care. Understanding the roles of MSLs in clinical trials, especially in procedure-driven fields like plastic surgery, has important implications for how clinical trials are conducted, interpreted, and applied in clinical practice. Improved integration and acknowledgment of MSLs can enhance scientific rigor and ultimately improve patient outcomes. To the best of our knowledge, this is the first scoping review to investigate the recognition and contribution of MSLs in plastic surgery clinical trials within the United States.

## Materials and Methods

This scoping review was conducted following the Preferred Reporting Items for Systematic Reviews and Meta-Analyses (PRISMA) 2020 guidelines, which are based on the guidelines of the Cochrane Handbook for Systematic Reviews of Interventions. The protocol for this review was preregistered on PROSPERO (CRD42023458751).

### Search Strategy

A search was performed for relevant studies on PubMed, Scopus, Cochrane Library, and ClinicalTrials.gov from their inception until June 2025. The complete database-specific search strings (including Boolean operators and filters) are provided in Supplementary Material 1 (available in the online version). No restrictions were placed on study design or publication date.

### Eligibility Criteria

Only plastic and reconstructive surgery-related clinical trials conducted in the United States that listed MSLs as authors or mentioned an MSL in the acknowledgment section were included. All participants in the trials evaluated were at least 18 years old. The primary objective was to identify the number of authors in clinical trial publications with the title of MSL. However, review articles, animal studies, and observational studies were excluded.

### Screening and Data Extraction

Two researchers independently scanned the titles, abstracts, and full texts of the references to select the studies to be included in this scoping review. Then, they independently extracted the data of interest from the included studies based on a preformed data extraction sheet. Any disagreement was resolved by discussion. To identify whether an author held an MSL role, we screened each article's full text, author affiliations, acknowledgment sections, and public records, including LinkedIn profiles and institutional webpages. If any ambiguity remained, designation was confirmed by triangulating institutional roles from external sources. We then documented whether MSLs were formally listed as co-authors or acknowledged in a dedicated section. This information was compiled to assess the extent to which MSLs were recognized in plastic surgery clinical trial publications, in alignment with the primary aim of the review.

### Quality Assessment

The NIH Quality Assessment Tool 7 was used to assess the risk of bias in the included studies. Discrepancies in the risk of bias assessment were resolved through consensus between the two reviewers. If consensus could not be reached, a third independent reviewer was available to serve as an arbitrator; however, no disagreements required escalation in this study.

### Analysis

**Fig. 1:**
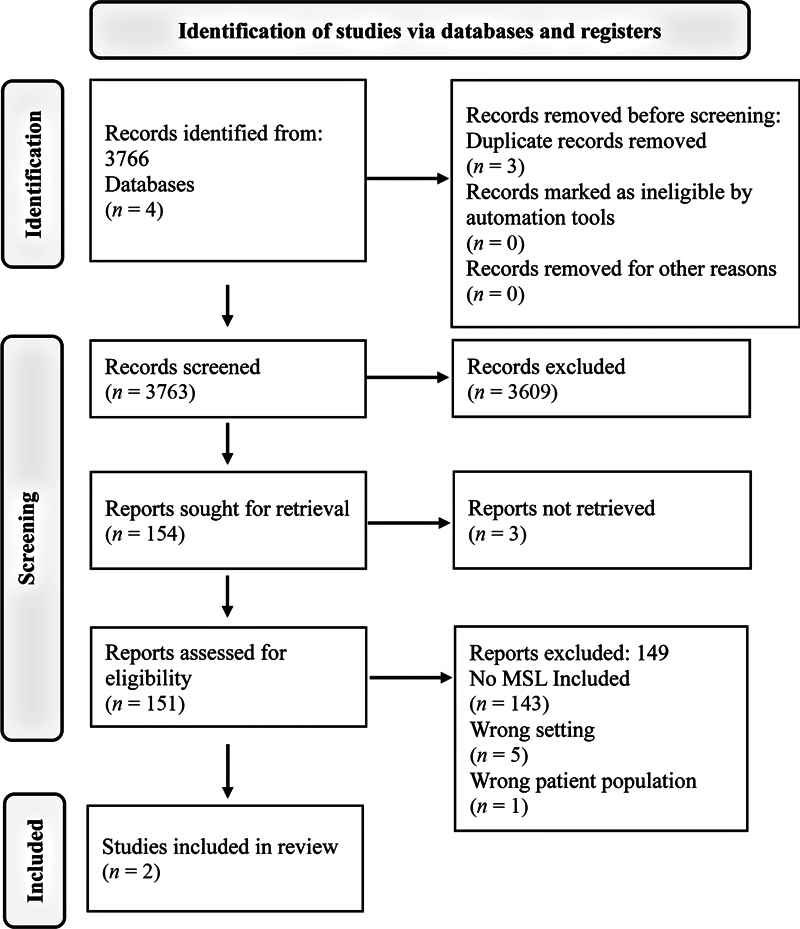
Flow diagram of the study identification and screening process, based on the PRISMA 2020 statement. This figure details the process of identifying, screening, and including studies from databases and registers, providing a transparent overview of how the final two studies were selected from the initial 3,766 records.

The lack of a detailed study precluded the use of a formal statistical analysis. Results were compiled, and a narrative synthesis was performed. A detailed overview of the study selection process is illustrated in Fig. 1 , following the PRISMA 2020 flow diagram format.

## Results

### Study Selection

The search strategy initially identified 3,766 studies. After duplicate removal and title/abstract screening, 154 plastic surgery clinical trials remained for full-text review. Of those, two studies met the inclusion criteria, with a total cohort of 452 patients and an average age of 40.8. Regarding quality assessment, the study by Smoot et al 8 was assessed as having a low risk of bias, while the study of Maxwell et al 9 was rated as having a moderate risk of bias, as summarized in Table 1 .

**Table 1:** Cochrane assessment risk

Study ID	The Cochrane Collaboration's tool for assessing risk of bias													
	Random sequence generation (selection bias)		Allocation concealment (selection bias)		Blinding of participants and personnel (performance bias)		Blinding of outcome assessment (detection bias)		Incomplete outcome data (attrition bias)		Selective reporting (reporting bias)		Other bias	
	Low/High/Unclear risk of bias	Reason	Low/High/Unclear risk of bias	Reason	Low/High/Unclear risk of bias	Reason	Low/High/Unclear risk of bias	Reason	Low/High/Unclear risk of bias	Reason	Low/High/Unclear risk of bias	Reason	Low/High/Unclear risk of bias	Reason
Smoot et al 8	Low		Low		Low		Low		Low		Low		Unclear	
Maxwell et al 9	High	Nonrandomized	High		High		High		Unclear		Unclear		Unclear	

### Summary of the Included Studies

This scoping review found that 2 out of the 154 studies leveraged MSLs. The first study, “Natrelle Style 410 Form-Stable Silicone Breast Implants: Core Study Results at 6 Years,” conducted by Maxwell and Van Natta, 9 was funded by Allergan, Inc., a global pharmaceutical company, and included MSL professionals in its authorship. Similarly, the study by Smoot et al, 8 financed by Pacira Pharmaceuticals, Inc., also featured authors with the title of MSL, utilizing compensated writing and editorial assistance supported by the company ( Table 2 ).

**Table 2:** Study characteristics

Study ID	Eligibility criteria		Results	Funding (private, public (grant), unknown, none)	Institution of first author	Institution of last author	Journal published
	Inclusion criteria	Exclusion criteria					
Maxwell et al 9	Women 18 years of age and older undergoing breast augmentation, reconstruction, or revision surgery with breast implants	They exclude women with precancerous conditions, active infections, pregnancy, health risks, unsuitable tissue, psychological issues, or unwillingness for future surgeries	While capsular contracture is a potential complication, the study suggests that using specific implants for some procedures might reduce the risk. Rupture rates were moderate, and most patients reported high satisfaction despite potential reoperations	Allergan USA, Inc. (private company)	Department of Plastic Surgery, Vanderbilt University School of Medicine	Plastic Surgery Associates and Medical Education Research Center; the Perkins Van Natta Center for Cosmetic Surgery and Medical Skincare; Allergan, Inc.; and the Department of Plastic Surgery, Vanderbilt University School of Medicine	Aesthetic Surgery Journal
Smoot et al 8	They included female patients 18 years of age or older who were not pregnant and had both breasts cosmetically enlarged under muscle using general anesthesia	The study excluded underweight patients, those having reconstructive surgery, and those recently been taking pain medications. Additionally, they excluded patients with other health issues, potential complications from the study drugs, or surgery-related complications that could affect their participation	The results suggest that DepoFoam bupivacaine could be a valuable new option for managing pain after breast augmentation surgery, but more research is needed to confirm this	Pacira Pharmaceuticals, Inc. (private pharmaceutical company)	Departments of Anesthesiology and Neurological Surgery, The Ohio State University, Columbus, Ohio, USA	Departments of Anesthesiology and Neurological Surgery, The Ohio State University, Columbus, Ohio, USA	Aesthetic Surgery Journal

### Maxwell et al 9

In the study conducted by Maxwell et al 9 titled “Natrelle Style 410 Form-Stable Silicone Breast Implants: Core Study Results at 6 Years,” the authors present updated results on the safety and effectiveness of the Natrelle Style 410 shaped, form-stable silicone gel implants over a period of 6 years. This prospective, nonrandomized, multicenter study included a diverse cohort of 941 patients categorized into four groups: 492 primary augmentations, 156 revision-augmentations, 225 primary reconstructions, and 68 revision-reconstructions. The focus was on evaluating the long-term clinical performance of these fifth-generation silicone gel implants.

Key findings demonstrated a relatively low complication rate across all patient groups. Capsular contracture (CC) rates, a common post-implant complication, were notably lower in the Natrelle 410 implant users compared with those with standard gel implants. Specifically, the 6-year CC rates were 4.6% for primary augmentations and 6.9% for revision-augmentations, which are significantly less than the rates observed with other implants. Furthermore, implant rupture rates were modest, with an overall rate of 6.4% per subject and 3.8% per implant across all cohorts.

### Smoot et al 8

In the randomized, double-blind study by Smoot et al, 8 published in the *Aesthetic Surgery Journal* , the efficacy and safety of DepoFoam bupivacaine were evaluated in patients undergoing bilateral, cosmetic, submuscular augmentation mammaplasty. This study was funded by Pacira Pharmaceuticals, Inc. The primary efficacy measure was the cumulative pain score with activity through 72 hours postoperatively, supplemented by secondary efficacy measures such as pain intensity, opioid consumption, and integrated rank analysis combining pain scores with opioid usage.

A total of 136 patients were treated and divided into two groups: DepoFoam bupivacaine ( *n* = 66) and bupivacaine HCl ( *n* = 70). The results showed that while the DepoFoam bupivacaine group had a lower mean cumulative pain score compared with the bupivacaine HCl group, the difference was not statistically significant, likely due to the early termination of the study, which left it underpowered. However, opioid consumption was significantly lower in the DepoFoam bupivacaine group at the 24- and 48-hour marks. Safety profiles were comparable between the two groups, with no serious adverse events reported.

The study suggests that DepoFoam bupivacaine may reduce opioid requirements postoperatively without compromising safety. Although the study was underpowered, DepoFoam bupivacaine shows potential as a valuable component of multimodal analgesia for breast augmentation surgery, pending further research to fully establish its efficacy and safety profile.

The inclusion of three MSLs in this study was acknowledged in the authorship list and aligns with typical MSL responsibilities as described in the literature. While the study did not formally assess the impact of MSLs on clinical outcomes, their involvement reportedly supported protocol execution, scientific communication, and field-level alignment between investigators and the sponsor. Based on the acknowledgments and author roles, MSL contributions likely included clarifying protocol requirements, ensuring study design feasibility in real-world clinical practice, and delivering ongoing scientific updates throughout the trial. These activities may have indirectly contributed to operational success, such as the observed lower opioid use in the treatment group; however, no causal relationship was examined or established in the original study. 3 4 Importantly, MSLs were not involved in drug development or regulatory decision-making but served as field-based medical experts who enhanced communication and protocol adherence. 3 6

## Discussion

Clinical trials are fundamental to medical research, providing essential evidence for the safety and efficacy of interventions. 2 10 Plastic surgery encompasses various procedures focused on restoring or enhancing form and function. This scoping review aims to explore the extent to which MSLs are acknowledged in published plastic surgery clinical trials within the United States and examines their specific roles and impact.

This scoping review identified 154 clinical trials in plastic surgery, of which only two studies acknowledged MSLs, suggesting they are underutilized due to a lack of standardized authorship criteria and limited formal integration into investigator-initiated trials. The first study, conducted by Maxwell et al, 9 included a cohort of 941 patients and evaluated the long-term clinical performance of Natrelle Style 410 silicone gel implants. The study found relatively low complication rates, including lower capsular contracture rates compared with other implants and high patient satisfaction rates. The second study by Smoot et al 8 investigated the efficacy and safety of DepoFoam bupivacaine in patients undergoing cosmetic breast augmentation surgery. DepoFoam bupivacaine demonstrated the potential to reduce opioid use postoperatively without compromising safety. Although causality cannot be determined from the available data, the inclusion of MSLs in these studies may have contributed to improved operational execution, including protocol adherence and investigator engagement, aligning with the review's objective to explore MSL impact. While neither study directly evaluated the relationship between MSL involvement and clinical outcomes, their acknowledged contributions to protocol support and communication are consistent with previously described MSL roles and suggest potential operational benefits worth further exploration. Although only two clinical trials met the inclusion criteria, the small number of studies explicitly acknowledging MSLs is an important finding. This highlights a systematic lack of transparency in reporting nonphysician scientific contributors, especially in plastic surgery trials, and indicates a broader issue in authorship practices across the medical research field. Scoping reviews, particularly in emerging or understudied areas, are essential in identifying existing gaps. In this case, the absence of data is meaningful because it suggests possible directions for future research and publishing practices.

Importantly, both studies utilized MSLs as part of their research teams and highlighted the critical role of MSLs in bridging the communication between clinical research and the pharmaceutical company they represent. 8 9 Notably, both studies were funded by pharmaceutical companies (Allergan and Pacira). While MSLs frequently contribute to various types of company-sponsored clinical research (i.e., Phase I–IV and investigator-initiated studies), their formal acknowledgment in publications is rare, as confirmed by previous research. There is no consistent framework for recognizing MSL contributions in trial reports or authorship, regardless of the type of clinical research. This review does not explicitly assess potential bias due to industry funding. Future studies should explore this aspect to better understand the potential influence of industry sponsorship on MSL involvement and study outcomes. Currently, no standardized tools or validated frameworks exist to quantitatively or qualitatively measure MSL contributions to clinical trials. This lack of assessment methodology represents a gap in the literature and a barrier to fully understanding and optimizing the scientific value MSLs bring to clinical research.

Regarding the studies of Smoot et al 8 and Maxwell et al, 9 MSL authors were included, and compensation writing assistance for the clinical trial was provided by the funding companies. Previous studies may not have explored the specific details of MSL contributions. 3 4 5 6 11

The majority of US-based MSLs support both company-sponsored and investigator-initiated clinical research, providing essential scientific expertise and facilitating effective communication and coordination, yet their contributions are often unrecognized. This scoping review revealed that MSLs are only acknowledged in a small percentage of plastic surgery clinical trials within the United States. This finding is consistent with previous studies across other medical specialties, 1 5 6 12 13 14 indicating a general lack of recognition of MSL contributions in trial publications. This lack of recognition not only undermines the contributions of MSLs but also represents a missed opportunity to highlight the diverse scientific expertise of all the professionals contributing to clinical trials, thereby enhancing the credibility of the trials.

Although the acknowledgment of MSLs in clinical research publications is limited, this review identified several significant activities of MSLs in plastic surgery clinical trials. These activities included providing scientific expertise and educational support, as well as facilitating communication between investigators and sponsoring pharmaceutical companies. Their involvement positively influenced critical success factors such as improved data quality. Some trials reported positive impacts attributed to MSL involvement, while others did not explicitly attribute any influence to MSLs. 3 4 5 6 11 15 16

MSLs are not limited to discussing and translating clinical research with physicians and HCPs; they are also significantly involved in recommending clinical trial sites to the sponsoring pharmaceutical company they represent. 3 6 11 12 13 The involvement of MSLs contributed to scientific rigor and consistency across trial sites. In Smoot et al, their support ensured alignment with clinical protocols and accurate interpretation of trial data, while in Maxwell et al, the MSL maintained continuous engagement with investigators, reinforcing protocol compliance and scientific transparency throughout the study. 3 4 5 6 11 12 13 16

## Strengths and Limitations

The strengths of this study include the following: (1) to the best of our knowledge, this is the first scoping review summarizing the acknowledgment of MSLs in plastic surgery clinical trial publications in the United States; (2) this review addresses a specific gap in the literature by focusing on the acknowledgment and roles of MSLs in plastic surgery clinical trials, providing targeted insights into this underexplored area; and (3) by investigating MSL acknowledgment and their potential impact, this review can enhance transparency and improve understanding of MSLs' contributions in plastic surgery clinical research. While the finding that MSLs are rarely acknowledged may appear intuitive to those familiar with the pharmaceutical industry, the implications extend into trial quality, regulatory alignment, and clinical applicability. MSLs contribute to key aspects of trial operations, protocol development, data interpretation, and scientific dissemination, which ultimately influence both the conduct and outcomes of clinical trials. Failure to recognize their involvement risks underrepresenting the interdisciplinary efforts required to deliver robust and clinically impactful research.

The limitations of this study include the following: (1) the scope of this review was limited to plastic surgery clinical trials conducted within the United States, which may not capture the full extent of MSL contributions in other geographical regions or medical specialties; (2) the identification of only 2 studies acknowledging MSLs out of 154 trials suggests a potential publication bias, where MSL contributions may be underreported or unacknowledged in many studies; and (3) the limited number of acknowledged MSLs and their specific roles in the identified studies restricts the generalizability of the findings, highlighting the need for more comprehensive reporting and acknowledgment of MSL activities in clinical trial publications.

This review explored the collaboration between MSLs, researchers, and plastic surgeons in clinical research. Building on this review and previous research, future investigations should expand the scope to include MSL contributions in clinical trials across various medical specialties and geographical regions, not just within the United States. This broader perspective can provide a more comprehensive understanding of the activities of MSLs in clinical research. In addition to expanding the research scope, future studies should focus on systematically evaluating the impact of MSLs on clinical research, such as investigator engagement, facilitating scientific exchange, and data interpretation. By addressing these areas, clinical researchers can better understand and leverage the expertise of MSLs, potentially leading to more robust and credible clinical trials. Future research should examine evaluation tools to measure MSL contributions, including protocol adherence, data quality, and investigator engagement or support. Additionally, establishing clear authorship guidelines for MSL participation in clinical trials may promote greater transparency and recognition of their contributions. Recognizing MSLs in publications not only validates their scientific contributions but also encourages greater interdisciplinary collaboration and transparency in clinical trial design, conduct, and reporting, particularly in specialties like plastic surgery, where nonphysician roles are often underrepresented. This scoping review was limited to two studies that explicitly acknowledged MSL contributions, and as such, we were unable to compare trials with and without MSL involvement. Future studies should aim to conduct such comparisons to better understand the relative impact of MSL participation on clinical trial outcomes.

## Conclusion

This scoping review highlights the significant yet often unrecognized contributions of MSLs in plastic surgery clinical trials conducted within the United States. Despite their critical roles in providing scientific expertise, facilitating effective communication, and ensuring data accuracy, MSLs are acknowledged in only a small fraction of these trials. This lack of recognition not only undermines their contributions but also represents a missed opportunity to enhance the credibility and success of clinical research. This scoping review confirms the scarcity of MSL acknowledgment in plastic surgery trial publications and emphasizes the need for recognition of the value and contribution of MSLs in clinical trials, particularly in plastic surgery within the United States. Further research is essential to fully understand and appreciate the impact MSLs have on clinical research and their role in advancing patient care. Recognition of MSLs in clinical trial publications enhances transparency, supports more accurate attribution of scientific contributions, and promotes collaborative research environments that ultimately strengthen the credibility and clinical relevance of trial findings.
