# Supplementary material for: Medical Science Liaisons in Clinical Trials for Plastic Surgery: A Scoping Review
Source: Indian J Plast Surg. 2025 Dec 30;59(1):15–21. doi: 10.1055/s-0045-1813038 (PMC13016848; doi:10.1055/s-0045-1813038)
Supplement: Supplementary file 1 — Supplementary Material [file 10-1055-s-0045-1813038-s2563566.pdf]

Supplementary Material 1. Full database search strategies for PubMed, Scopus, Cochrane Library, and ClinicalTrials.gov (searches updated through June 2025).

((((plastic[tw] OR cosmetic[tw] OR reconstructi*[tw] OR “aesthetic”[tw] OR hand[tw] OR “Craniofacial”[tw] OR “burn”[tw]) AND (surger*[tiab])) OR ((“Reconstructive Surgical Procedures”[Mesh]) OR (“Surgery, Plastic”[Mesh]) OR “microsurgery”[tw])) AND ((United States [mh] OR “united states” OR usa OR u.s.a. OR Appalachia* OR “great lakes” OR mid-atlantic-state* OR mid-atlantic-region* OR middle-atlantic-state* OR middle-atlantic-region* OR midwestern-us* OR midwestern-u.s* OR Midwestern-state* OR Midwest-state* OR Midwest-us* OR Midwest-u.s* OR “great plains” OR heartland OR “new england” OR northeastern-us* OR northeastern-u.s* OR northeastern-state* OR northeast-state* OR northeast-us* OR northeast-u.s* OR “pacific northwest” OR northwestern-us* OR northwestern-u.s* OR northwest-u.s* OR northwest-us* OR northwestern-state* OR northwest-state* OR pacific-state* OR southeast-state* OR southeastern-state* OR southeast-region OR southeastern-region OR southeast-us* OR southeastern-us* OR southeast-u.s* OR southeastern-u.s* OR southern-state* OR southern-us* OR southern-u.s* OR southwest-state* OR southwestern-state* OR southwest-us* OR southwestern-us* OR southwest-u.s* OR southwestern-u.s* OR “deep south” OR “black belt” OR “rust belt” OR “district of Columbia” OR “Washington dc” OR Washington-d.c. OR Alabama OR (Birmingham [ad] AND al [ad]) OR Huntsville [ad] OR (Montgomery [ad] AND al [ad])OR Alaska OR anchorage [ad] OR fairbanks [ad] OR Arizona OR Phoenix [ad] OR Tuscon [ad] OR Flagstaff [ad] OR Arkansas OR “little rock” OR California OR “los angeles” OR “san diego” OR “san Francisco” OR Berkeley [ad] OR Stanford [ad] OR Colorado OR Vail [ad] OR Denver [ad] OR Connecticut OR Farmington [ad] OR “new haven” [ad] OR Hartford [ad] OR Delaware OR Wilmington [ad] OR Newark [ad] OR Florida OR Miami [ad] OR Gainesville OR Jacksonville OR Tampa OR Tallahassee OR Georgia OR Atlanta OR (Athens [ad] AND ga [ad]) OR (Augusta [ad] AND ga [ad]) OR Hawaii OR Hawai'i OR Honolulu OR Idaho OR Boise [ad] OR Illinois OR Chicago OR Urbana [ad] OR Evanston [ad] OR Indiana OR Indianapolis OR “West Lafayette” OR Iowa OR Kansas OR Wichita OR Kentucky OR Lexington [ad] OR Louisville [ad] OR Bardstown [ad] OR Louisiana OR “new Orleans” OR “baton rouge” OR Shreveport OR Maine OR Orono OR (Scarborough [ad] AND me [ad]) OR Maryland OR Bethesda [ad] OR Baltimore [ad] OR Rockville [ad] OR “johns Hopkins” OR Massachusetts OR Boston OR Harvard OR (worcester [ad] AND ma [ad]) OR Burlington [ad] OR Michigan OR Detroit OR “ann arbor” OR “east lansing” OR Minnesota OR Minneapolis OR Rochester OR “st paul” [ad] OR “saint paul” [ad] OR Mississippi OR (Jackson [ad] AND ms [ad]) OR Missouri OR (Columbia [ad] AND mo [ad]) OR Montana OR Bozeman [ad] OR Missoula OR Nebraska OR Omaha [ad] OR Lincoln [ad] OR Nevada OR “Las Vegas” OR “New Hampshire” OR “New Jersey” OR “New Mexico” OR “New York” OR “North Carolina” OR “North Dakota” OR Ohio OR Columbus [ad] OR Cleveland [ad] OR Cincinnati OR Oklahoma OR Oregon OR Portland [ad] OR Pennsylvania OR Philadelphia OR Hershey [ad] OR “Rhode Island” OR providence [ad] OR “South Carolina” OR “South Dakota” OR Tennessee OR Nashville OR Memphis OR Texas OR Houston OR Utah OR Vermont OR Virginia OR Richmond [ad] OR Washington [tiab] OR Washington [ad] OR Seattle OR “West Virginia” OR Wisconsin OR Wyoming))) AND (“randomized trial” OR “clinical trial” OR “parallel trial” OR “crossover trial” OR “sequential trial” OR “open label” OR “blind study” OR “superiority trial” OR “non-inferiority trial” OR “non inferiority trial” OR “nonrandomized” OR “controlled trial” OR “uncontrolled trial” OR “Clinical Trial” [Publication Type]) AND

((2012:2022[pdat]))) NOT (“Review” [Publication Type] AND “Review Literature as Topic”[Mesh] AND (english[Filter]))
